# Use of synthetic slings in the treatment of female stress urinary incontinence: Number 2 – 2025

**DOI:** 10.61622/rbgo/2025FPS2

**Published:** 2025-03-17

**Authors:** Marair Gracio Ferreira Sartori, Marilene Vale de Castro Monteiro, Cássia Raquel Teatin Juliato, Luiz Gustavo Oliveira Brito, Sergio Brasileiro Martins, José Miguel de Deus, Ana Selma Bertelli Picoloto, Jorge Milhem Haddad, Andreisa Paiva Monteiro Bilhar, Leticia Maria de Oliveira, Rafael Mendes Moroni, Lucas Schreiner, Aljerry Dias do Rego, Daniela Siqueira Prado, Emerson de Oliveira

**Affiliations:** Universidade Federal de São Paulo Escola Paulista de Medicina São Paulo SP Brasil Escola Paulista de Medicina, Universidade Federal de São Paulo, São Paulo, SP, Brasil; Universidade Federal de Minas Gerais Belo Horizonte MG Brasil Universidade Federal de Minas Gerais, Belo Horizonte, MG, Brasil; Universidade Estadual de Campinas Campinas SP Brasil Universidade Estadual de Campinas, Campinas, SP, Brasil; Universidade Estadual de Campinas Campinas SP Brasil Universidade Estadual de Campinas, Campinas, SP, Brasil; Universidade Federal de São Paulo Escola Paulista de Medicina São Paulo SP Brasil Escola Paulista de Medicina, Universidade Federal de São Paulo, São Paulo, SP, Brasil; Universidade Federal de Minas Gerais Belo Horizonte MG Brasil Universidade Federal de Minas Gerais, Belo Horizonte, MG, Brasil; Universidade Federal do Rio Grande do Sul Porto Alegre RS Brasil Universidade Federal do Rio Grande do Sul, Porto Alegre, RS, Brasil; Universidade de São Paulo São Paulo SP Brasil Universidade de São Paulo, São Paulo, SP, Brasil; Universidade Federal do Ceará Fortaleza CE Brasil Universidade Federal do Ceará, Fortaleza, CE, Brasil; Universidade Federal de São Paulo Escola Paulista de Medicina São Paulo SP Brasil Escola Paulista de Medicina, Universidade Federal de São Paulo, São Paulo, SP, Brasil; Universidade Estadual do Oeste do Paraná Cascavel PR Brasil Universidade Estadual do Oeste do Paraná, Cascavel, PR, Brasil; Pontifícia Universidade Católica do Rio Grande do Sul Porto Alegre RS Brasil Pontifícia Universidade Católica do Rio Grande do Sul, Porto Alegre, RS, Brasil; Universidade Federal do Amapá Macapá AP Brasil Universidade Federal do Amapá, Macapá, AP, Brasil; Universidade Federal de Sergipe São Cristóvão SE Brasil Universidade Federal de Sergipe, São Cristóvão, SE, Brasil; Faculdade de Medicina do ABC Santo André SP Brasil Faculdade de Medicina do ABC, Santo André, SP, Brasil

## Key points

- Stress urinary incontinence (SUI) is a very common condition in women and negatively affects quality of life.
- Treatment of SUI should begin with behavioral and clinical measures.
- Surgical treatment should be indicated when clinical treatment fails or at the patient's discretion after being informed about the therapeutic possibilities.
- Several surgical techniques have been described for treatment, but the gold standard procedure is represented by mid-urethral slings.
- Synthetic mid-urethral slings have been questioned due to the complications observed with synthetic meshes used to treat genital prolapse.

## Recommendations

- Synthetic mid-urethral slings available on the Brazilian market are safe and effective in the treatment of SUI.
- The synthetic material used in slings is the macroporous monofilament polypropylene.
- Surgeries with synthetic slings should be performed by technically trained surgeons with knowledge of how to avoid and treat potential complications.
- The patient should be informed about the benefits and possible risks of using synthetic material.
- If the patient does not wish to use synthetic material, other options should be proposed, such as Burch colposuspension or autologous pubovaginal sling.
- Urethrocystoscopy should always be performed on retropubic slings and, when possible, on other slings.
- Synthetic slings are considered the gold standard for the surgical treatment of SUI.

## Background

Stress urinary incontinence (SUI) in women is defined as any involuntary loss of urine that occurs during exertion, such as coughing or sneezing.^(1)^

The prevalence of SUI depends on the definition used. Most adult women report isolated episodes of small losses sporadically, without clinical harm. However, severe and frequent losses are reported by approximately 10% of women aged between 40 and 50 years, plus it is well known that the prevalence increases with advancing age.^(2)^ In Brazil, the estimated prevalence of urinary incontinence after the fourth decade of life is 25%.^(3)^

The quality of life of women with SUI is significantly affected, even when the losses occur in small quantities. Patients with incontinence suffer limitations in the physical, social, psychological and even financial spheres. They stop participating in social activities by feeling shame and embarrassment, in addition to increasing expenses with pads, medicines and medical care. Furthermore, not all women know that urinary incontinence is a disease, as they believe this is part of the aging process, and around 30% of them seek treatment. Therefore, highly effective and low morbidity treatments are crucial for greatly improving self-esteem and quality of life.^(4)^

There is a consensus that conservative treatment should always be offered to patients. Functional rehabilitation of the pelvic floor and behavioral changes are encouraged, and surgical treatment should be offered when clinical treatments fail.^(5)^

Therefore, it is important to discuss the possibilities of surgical treatment, its indications, contraindications, results and complications, so these procedures can be indicated and performed responsibly and safely. For many years, the technique of vaginal plication of the bladder neck, known as the Kelly-Kennedy surgery, was used, which included plication of the vesicovaginal fascia. This technique has been abandoned because the long-term success rates are low and it leads to periurethral fibrosis. Pubourethral sling surgeries with autologous material date back to the 1950s, and retropubic colpofixation using the Burch technique dates back to 1961. The aim of both surgeries is to reposition the bladder neck in an intrabdominal position. They are based on the theory of bladder neck hypermobility as the genesis of SUI, according to which the difference in pressure between the urethra and the bladder during times of exertion would lead to urine loss. Hence, repositioning the bladder neck would allow an equal distribution of abdominal pressure across the bladder and bladder neck, preventing urine loss.^(5)^

A new theory, known as the Integral Theory, was proposed in 1990, advocating that urine loss occurs due to loss of urethral support, particularly due to injury to the pubourethral ligaments.^(6)^ As a result, the first synthetic mid-urethral slings, commercially known as TVT (tension-free vaginal tape), were described. This type of surgery quickly became widespread and used worldwide in the minimally invasive treatment of SUI.^(7)^

Complications such as bladder, bowel, and large vessel perforation began to appear with the increasing number of surgeries performed. Thus, in the 2000s, another synthetic sling technique using the transobturator route was developed, thus avoiding the retropubic space. More recently, single-incision slings have also been launched in an attempt to reduce complications, the size of the synthetic strip and the operative time.^(8)^

In 2008, due to serious complications related to synthetic meshes used in the treatment of genital prolapse via the vaginal route, the FDA (Food and Drug Administration) issued the first warning about the use of these devices. New warnings were issued later, culminating in the withdrawal of numerous synthetic systems for genital prolapse from the market in several countries.^(9)^

Although the FDA maintained the marketing authorization for synthetic slings for the treatment of SUI, several countries suspended the marketing of these devices.^(10)^

Mid-urethral slings are currently the most commonly performed surgery for the treatment of SUI around the world with an estimated 250,000 surgeries performed in the United States alone in 2010.^(10)^

In Brazil, synthetic slings for the treatment of SUI continue to be available. Therefore, the National Specialized Committee on Urogynecology of Febrasgo prepared this document based on evidence with high levels of recommendation to guide the use of synthetic slings in Brazil.

## Is the material used to manufacture synthetic slings safe?

Polypropylene has been used as a suture thread for over 50 years in surgical procedures in a wide range of medical specialties. The polypropylene used in the manufacture of sling strips is braided and designed to minimize the risk of infection, exposure, and fibrosis. As a result, significant differences in the biomechanical properties between sling straps and meshes for abdominal hernia repair are observed, and slings are lighter due to their macroporous characteristics.^(11)^

Compared to slings with meshes, monofilament slings are more durable, safe, and effective, with consistent studies and long follow-up.^(12-13)^

The use of synthetic slings made of polypropylene monofilament with macropores mesh, known as Type 1 meshes, is recommended, as they are considered safe and effective in the treatment of SUI.

## Is it possible to use manufactured synthetic meshes instead of commercial sling kits?

Some authors report the use of cut Type 1 polypropylene meshes to manufacture slings for cost reduction, thereby making the treatment available to a greater number of women.^(14-16)^ However, there are few studies with small sample sizes and short follow-up.

As scientific evidence is lacking, the use of manufactured synthetic meshes is not recommended in the surgical treatment of SUI.

## What are the necessary qualifications of the surgeon to perform mid-urethral synthetic sling procedures?

All surgical procedures require adequate training of the surgeon both in the surgical technique and in the management of intra- and postoperative complications.^(17)^

According to recommendations of the manufacturer of the original TVT, the surgeon should have experience in urethrocystoscopy, extensive anatomical knowledge of the retropubic space, have participated in or assisted in five surgeries, and performed another five surgeries under the supervision of an experienced surgeon, strictly following the device's instructions. Most studies evaluating the learning curves for mid-urethral slings indicate that it takes approximately 8-30 procedures to reduce complications for experienced surgeons, and 20-50 for surgeons in training.^(18)^

Although the greater the surgeon's experience, the greater the tendency to increase the success rates and reduce complications of slings, no differences in the incidence of complications between urologists and gynecologists have been observed. Adequate training in the procedure and management of complications is essential.^(19)^

As there is no consensus on the number of surgeries performed, it is recommended that the surgeon is able to perform slings if he/she:

- has experience in diagnosing SUI;
- knows how to indicate the most appropriate procedure and is able to explain it to the patient so that she can give an opinion on her treatment;
- has adequate knowledge of the pelvic anatomy, particularly the areas with a higher or lower risk of injury;
- has demonstrated prior supervised surgical training and experience in performing the procedure safely and effectively;
- is able to perform urethrocystoscopy;
- is aware of the potential complications and can explain them to patients;
- has the ability to diagnose and treat potential complications.

## What are the success rates of mid-urethral slings?

Although follow-up studies of over ten years of mid-urethral slings show a cure rate ranging from 62% to 87%, the cure rate decreases over time.^(20)^ Retropubic slings and autologous slings have the highest cure rate, followed by transobturator slings.^(13,21,22)^

Subjective cure rates can range from 43% to 92% in transobturator slings and from 51% to 88% in retropubic slings, and the average in both groups is 88%, according to Ford et al. (2017).^(20)^ In this same systematic review, an objective cure rate of 85.7% was observed for transoburatory slings and of 87.2% for retropubic slings.^(20)^

## What are the most frequent complications of mid-urethral slings and their incidence?

Complications with the use of slings are low, with readmission and reoperation rates ranging from 0.6% to 0.8%. The main complications are listed in chart 1^(23)^

## Pain

Pain or discomfort in the groin or inner thigh region, regardless of intensity, occurs mainly after transobturator slings (RR: 4.12; 95% confidence interval [CI]: 2.71-6.27). However, the intensity of suprapubic pain is significantly lower with transobturator slings (RR: 0.29; 95% CI: 0.11-0.78). Most pain (80%-90%) resolves within the first six months after the procedure, with an average of eight weeks.^(20)^

## Sling exposure or extrusion and organ perforation

Sling exposure is defined as the visualization of the synthetic strip separated from the vaginal mucosa. Extrusion, in turn, refers to the passage of the mesh forming a loop in the vaginal mucosa, vagina or urethra. Perforation means an abnormal opening of a hollow organ or viscera.^(24)^ Note that the exposure of the mesh in the vaginal wall does not cause symptoms in most patients, therefore, it does not alter the quality of life.

The extrusion rates of synthetic slings are rare, occurring in less than 1% of cases. Vaginal exposure is more common in transobturator slings (up to 10%), and bladder perforation occurs more frequently in retropubic slings (up to 24%).^(23)^

## Voiding dysfunction and overactive bladder

Irritation symptoms such as urgency and increased urinary frequency are present in up to 25% of operated patients.^(23,25,26)^ In a systematic review of 3,139 women, the incidence of storage symptoms after slings was around 9%, regardless of the type of sling.^(20)^ There was also no significant difference in long-term storage symptoms or presence of detrusor overactivity between retropubic and transobturator slings.^(20)^

## Urinary retention

In general, urinary retention after slings is temporary and self-limited.^(27)^ Transobturator slings have lower rates of postoperative urinary dysfunction than retropubic slings (RR: 0.53; 95% CI: 0.43-0.65).^(20)^

## Infections

Urinary tract infections (UTIs) and surgical site infections are the most common complications after sling surgeries.^(28)^ The main risk factors for UTI after sling surgeries are: association with pelvic floor reconstruction and hysterectomies, duration of surgery, frequent manipulation with a cystoscope, urethral catheter during the procedure, hematomas at the surgical site, bladder perforation, postoperative retention, and indwelling or intermittent catheterization.^(29)^ The rate of UTI after sling surgery can reach 34% in the first three months after surgery and up to 50% after one year.^(29)^ Other risk factors are advanced age and diabetes. The earlier the urinary catheter is removed, the lower the rate of postoperative UTI after sling surgery.^(29)^

## Dyspareunia

In several studies, dyspareunia (internal vaginal pain during or after intercourse) is assessed together with pelvic pain and pain at the root of the thigh, making it difficult to assess it separately. This symptom is more frequent after synthetic slings than in autologous slings. There is no consistent evidence that it is more frequent in transobturator slings than in retropubic slings.^(20,30)^

When dyspareunia occurs prior to sling surgery, there may be resolution or improvement of approximately 6% to 73%, probably due to reduced urinary incontinence during intercourse and improved quality of life.^(31)^ New dyspareunia may result from exposure of the sling mesh (regardless of the retropubic or transobturator route) and the presence of a subepithelial foreign body without exposure.

## Vascular injury

Large vessel injury is rare, and retropubic hematoma is the most common. It is more prevalent after retropubic slings than after transobturator slings.^(20)^ Vascular injury can occur in between 0.5% and 2.5% of sling surgeries and is most often of venous origin (later diagnosis) rather than arterial (earlier diagnosis with greater severity). The most frequently affected vascular structures are the periurethral vascular plexus, inferior epigastric vessels, external iliac vessels, and obturator vessels.^(32)^

Early recognition of vascular injury improves the prognosis and prevents serious consequences for the patient, and appropriate training of the surgeon is important.

## Fistulas

The occurrence of vesicovaginal, urethrovaginal, or vesicocutaneous fistulas is extremely rare after mid-urethral slings. In general, fistulas are caused by unidentified exposures, extrusions or perforations.^(33)^

The risk factors for fistulas after sling are the same as for mesh exposure or extrusion: previous pelvic surgery, greater tension of the sling mesh under the urethra, inadequate surgical dissection, extensive atrophy of the vaginal walls and previous pelvic irradiation.^(34,35)^

The time between sling surgery and the diagnosis of fistulas can vary from ten to 36 months, but there are reports of appearance up to eight years later.^(34)^ The main symptoms are urinary incontinence, bladder hyperactivity, symptoms of voiding dysfunction or pelvic pain.

## Are the results of slings different from those of procedures without synthetic material?

Mid-urethral slings have higher cure rates (82%) compared to retropubic colposuspension (Burch technique, 74%), although with a higher risk of vaginal and bladder perforation.^(36)^

Mid-urethral slings have similar efficacy and complication rates to those of slings without the use of synthetic material (autologous pubovaginal slings). However, pubovaginal slings have a higher incidence of urinary storage symptoms and a higher risk of needing reoperation than slings made of synthetic material.^(37)^ The main advantage associated with pubovaginal slings is the lower risk of exposure, as they have minimal inflammatory reaction. The disadvantages of pubovaginal slings include longer surgical time and higher risk associated with the surgical site for fascia removal, such as bleeding and infection.^(37)^

## Are the complications of slings different from those of procedures without synthetic material?

Sling surgeries can be performed via the retropubic or transobturator route, using either synthetic or autologous material. Different needles may be used, regardless of the route, but the complications are similar.^(38)^ Mesh exposure, refractory pelvic pain, and dyspareunia are more common after synthetic slings.^(38)^ Abdominal wall infections, retropubic hematomas, bladder hyperactivity, and urethral obstruction requiring further surgery are more common after autologous slings.^(39)^

## When should synthetic slings not be used?

The use of a synthetic sling is not recommended if the urethra has been inadvertently injured during the procedure. Sling placement is also not indicated if the surgical procedure includes correction of a urethral diverticulum or urethral fistula. The use of synthetic meshes should be avoided in patients at risk of poor healing, such as after irradiation, with many previous scars or evidence of poor quality tissue.^(40)^ The insertion of slings in immunosuppressed patients requires greater care given the greater risk of infection.

## Should urethrocystoscopy be considered for sling surgeries?

Although bladder or urethral perforations in sling surgeries are rare, early identification of these injuries allows immediate correction and avoids late complications, reinforcing the importance of intraoperative urethrocystoscopy. On the other hand, performing urethrocystoscopy increases surgical time and requires specialized training.

Since urethrocystoscopy is a low-risk procedure that can detect potential complications, there is a consensus that it should be performed routinely in retropubic procedures, in which the risks of bladder injury are greater.^(41-43)^ Therefore, it is recommended to perform intraoperative cystoscopy in cases of retropubic slings and, whenever possible, in other slings.

## Final considerations

Several international urogynecology societies have issued statements in favor of the use of synthetic slings in the treatment of SUI.^(44-46)^ Since their launch, synthetic slings have been the most studied type of surgery and have received the highest number of publications in scientific journals with a rigorous selection process. It is the most well-studied surgery for the treatment of SUI of all time, with more than 2,000 publications, showing high efficacy and a low complication rates.

There are studies on slings in various populations worldwide, including healthy women or those with comorbidities, young and older adult women, in cases of mild, moderate or severe loss, in primary or relapse cases and in different ethnic groups. There are also comparative studies between synthetic slings and other surgical techniques for SUI demonstrating high efficacy and safety.

Thus, synthetic slings inserted by qualified surgeons in patients with a specific indication are safe and effective, and should be considered the gold standard for the treatment of SUI in women.

## National Specialized Commission on Urogynecology and Vaginal Surgery of the Brazilian Federation of Gynecology and Obstetrics Associations (Febrasgo)

**President:**

Marair Gracio Ferreira Sartori

**Vice-president:**

Cassia Raquel Teatin Juliato

**Secretary:**

Marilene Vale de Castro Monteiro

**Membros:**

Aljerry Dias do Rego
  Ana Selma Bertelli Picoloto
  Andreisa Paiva Monteiro Bilhar
  Daniela Siqueira Prado
  Emerson de Oliveira
  Jorge Milhem Haddad
  Leticia Maria de Oliveira
  Lucas Schreiner
  Luiz Gustavo Oliveira Brito
  Rafael Mendes Moroni
